# The Effect of Fig Tree Latex (Ficus carica) on Stomach Cancer Line

**Published:** 2011-04-01

**Authors:** S A Hashemi, S Abediankenari, M Ghasemi, M Azadbakht, Y Yousefzadeh, A A Dehpour

**Affiliations:** 1 Department of Microbiology and Immunology, Mazandaran University of Medical Sciences, Sari, Iran; 2 Department of Pathology, Mazandaran University of Medical Sciences, Sari, Iran; 3 Department of Pharmacogenosy, Mazandaran University of Medical Sciences, Sari, Iran; 4 Department of Biology, Islamic Azad University, Ghaemshahr Branch, Ghaemshahr, Iran

**Keywords:** Fig, Ficus carica, Stomach cancer cell line, Latex

## Abstract

**Background:**

The therapeutic effect of herbal materials in inhibition of cancer cell growth was shown. This study investigates the effect of fig tree latex (Ficus carica) on stomach cancer line.

**Methods:**

The in vitro effect of different doses of fig tree latex on stomach cancer cell line and the peripheral blood mononuclear cells was evaluated after 72 hours.

**Results:**

Fig tree latex could inhibit the proliferation of cancer cell line without any cytotoxic effect on human normal cells. Five mg/ml was the optimum concentration in inhibition of cell line growth.

**Conclusion:**

Cancer cell line was more sensitive to Ficus carica latex than normal cells. This anticancer activity might be due to presence of its proteolytic enzymes.

## Introduction

Cancer originates from some mutations in transformed cells and other heritable variations in susceptible cells. So far, abnormalities in about 350 genes have been demonstrated in human cancers[1][2] and epidemiologically, cancer is responsible for one in eight of worldwide deaths.[3] Gastrointestinal tract cancers are considered as the most important causes of global deaths. In 2000, [2][3] million of cancer cases were in alimentary tract presenting in pharynx, oesophagus, stomach and colorectal region. In Fars Province, southern Iran, the annual crude incidence rate and ASR regarding cancer of stomach was reported 2.32 and 3.82 in males while these figures in females were 1.03 and 1.60.[4]

It was shown that neoplasias in digestive organ are mostly due to modification in dietary habits,[5] and in this relation, plants and herbals as natural products were reported to have anti-cancer properties and even play an important role in the efficacy of chemotherapy. [6][7] Different parts of Ficus carica (fig) and Ficus sycomorus were studied as herbal medicine. Latex is a substance originating from young leaves of fig tree when broken[8] and has a cysteine proteinase enzyme that is active in a pH range of 6.5-8.5.[9] Injection of Ficus carica latex was shown to change the growth rate of a benz- [a]-pyrene-induced sarcoma and could suppress small tumors in albino rats.[10] Antioxidants and polyphenolic properties of fruits were demonstrated as an anti-inflammatory activity of fruits.[11][12][13][14]The reports show that polyphenolic component of fruits has an antioxidant, antiinflammatory, antialergic, antimicrobial and anticancer effect.[15][16] Ficus carica latex and its derivatives have been shown to suppress the growth of transplanted and spontaneous tumors in mice.[17][18] Therefore in the present study, the therapeutic effect of fig tree latex on stomach cancer cell line and peripheral blood mononuclear cells were investigated in vitro.

## Materials and Methods

We collected Ficus carica (fig) latex from fig tree in Sari (Iran) drop-by-drop through cutting young leaves of fig tree. Different concentrations of latex were provided including 0.125, 0.25, 0.5, 1, 2.5 and 5 mg/ml. The stomach cancer cell line was provided from National Cell Bank of Iran, NCBI=C-131. 3ҳ104 cells were cultured in liquid medium (RMPI 1640) containing 10% fetal calf serum, 100 U/L penicillin and streptomycin. The culture flask’s environment was kept at 37°C, with a saturated humidity and 5% CO2.

A peripheral heparinized blood sample was collected from 3 normal subjects and the mononuclear cells were isolated by centrifugation on a Ficoll histipaque (1.077, Sigma, USA). The cells from the interphase were washed three times with RPMI (1640 medium, Gibco) and counted and their viability was determined by trypan blue. All samples were run triplicates in 96-well plates. Cultures were incubated at 37°C in a humidified 5% CO2 atmosphere for 3 days and then pulsed with 200 μl 3-[4,5- dimethylthiazolyl]-2,5-diphenyl-tetrazolium bromide (MTT: Sigma) as a color indicator of metabolic activity. The supernatant was harvested for 4 hrs. Later, dimethylsulfoxide (DMSO) was added (200 μl) and the color change was read in an ELISA reader at 630 nm wave length.[19] Data are presented as mean±SD. For statistical analysis, paired t-test was used.

## Results

**Fig. 1 :**
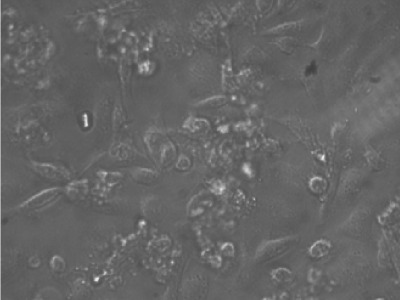
Growth of stomach cancer cell line without treatment of Ficus carica latex after 72 h

**Fig. 2 :**
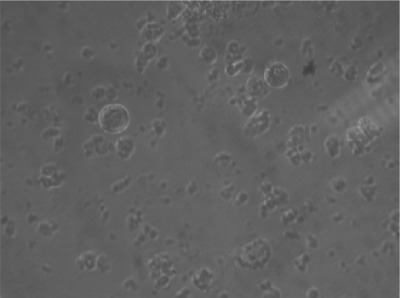
Growth inhibition of stomach cancer cell line treated with Ficus carica latex (5 mg/ml) after 72 h.

**Fig. 3 :**
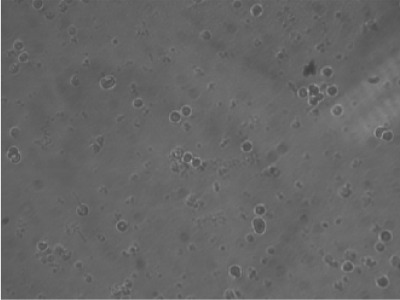
Peripheral blood mononuclear cells treated with Ficus carica latex (5 mg/ml) after 72 h.

After 72 h treatment of 1 mg/ml concentration of Ficus carica latex in culture media, the mean±SD was 0.23±0.033. In addition, for 2.5 mg/ml was 0.183±0.04 and in the concentration of 5 mg/ml, the mean±SD reached 0.17±0.014. The proliferation level of 1, 2.5 and 5 mg/ml concentrations of Ficus carica latex were significantly different from the control (Table 1). After 72 h incubation, the effects of various concentrations of Ficus carica latex on cancer and normal cells were presented in Figure 1, Figure 2 and Figure 3.

**Table 1 :** The effect of Fig tree latex on stomach cancer cell line proliferation in comparison with control in culture media, evaluated by MTT assay (optical density of 630 nm).

**Fig concentration (mg/ml)**	**Mean±SD**	**P value**
0.125	0.2687±0.06529	0.085
0.25l	0.2913±0.08109	0.40
0.5	0.3020±0.09627	0.463
1 l	0.2323±0.03398	0.033
2.5l	0.1830±0.04051	0.025
5	0.1763±0.01498	0.008
control	0.3567±0.02743	

## Discussion

In this research, the anticancer effect of Ficus carica latex in different concentrations was studied. The 5 mg/ml concentration had the greatest effect in inhibition of stomach cancer cell line growth but without any obvious effect on peripheral blood mononuclear cells. Wang et al.[20] studied the effect of fresh fig fruit latex on human cancer cell line and showed that the fresh latex acted as an anticancer substance. In contrast with our work, the dried form of Ficus carica latex was used and after 3 months, it was weighted and solved it in 1 ml of distilled water. After filtration, it was used in different concentrations. It was shown that fig tree latex powder can save its anticancer properties after a long period of time and may be used as an anticancer substance.

Ficin,[9] a cysteine proteinase isolated from the latex of Ficus carica tree is known to occur in various forms. Cysteine proteinases are a group of enzymes leading to apoptosis of cancer cells.[21] Furthermore, the anticancer effects may be associated with antioxidant properties11 due to its polyphenolic components.[12][13][14] Additionally, Ficus carica latex inhibited the proliferation of cancer cell line but did not indicate any cytotoxic activity against normal cells in vitro. We concluded that Ficus carica can have an anticancer cell activity while cancer cells were more sensitive to this latex than normal cells.
